# The association between blood lipids and cognitive impairment in type 2 diabetes mellitus

**DOI:** 10.1186/s40001-023-01574-w

**Published:** 2024-01-02

**Authors:** Li Ma, Yue-Xing Yuan, Feng-Jin Cheng, Yan Liu, Qiong Wei, You-Fan Peng, Yao Wang

**Affiliations:** 1 Department of Rehabilitative Medicine, Affiliated Zhongda Hospital of Southeast University, Nanjing, 210009 Jiangsu China; 2 Department of Endocrinology and Metabolism, Affiliated Zhongda Hospital of Southeast University, Nanjing, 210009 Jiangsu China; 3 Department of Rheumatology and Immunology, The Affiliated Drum Tower Hospital of Nanjing University Medical School, Nanjing, 210009 Jiangsu China; 4 Zhongda Hospital, Institute of Diabetes, School of Medicine, Southeast University, Nanjing, 210009 Jiangsu China; 5 Department of Respiratory and Critical Care Medicine, The Affiliated Hospital of Youjiang Medical University for Nationalities, Baise, 533000 Guangxi China

**Keywords:** Type 2 diabetes mellitus, Blood lipids, Cognitive impairment

## Abstract

**Objective:**

The study was performed to explore the association between blood lipids and cognitive impairment in patients with type 2 diabetes mellitus** (**T2DM).

**Methods:**

This study included 336 patients with T2DM. Relevant clinical data including total cholesterol (TC), high-density lipoprotein cholesterol (HDL-C), low-density lipoprotein cholesterol (LDL-C), triglyceride (TG), apolipoprotein A1, apolipoprotein B were collected, and the Mini-Mental State Examination (MMSE) score and Montreal Cognitive Assessment (MoCA) score were used to assess the cognitive function in patients with T2DM.

**Results:**

Serum apolipoprotein A1 levels were significantly increased in T2DM patients with cognitive impairment compared with T2DM patients without cognitive impairment (*p* = 0.017). Serum apolipoprotein A1 levels were significantly negatively correlated with MoCA score (*r* = − 0.143, *p* = 0.009) and MMSE score (*r* = − 0.132, *p* = 0.016) in patients with T2DM. In multivariable-adjusted regression model, serum apolipoprotein A1 was independently associated with cognitive impairment in patients with T2DM (OR = 5.201, *p* = 0.024).

**Conclusion:**

Serum apolipoprotein A1 is associated with cognitive impairment in patients with T2DM, but not TC, TG, HDL-C, LDL-C, and apolipoprotein B, indicating that increased serum apolipoprotein A1 may be a risk factor of cognitive impairment in patients with T2DM.

## Introduction

Type 2 diabetes mellitus (T2DM) is associated with various chronic complications, such as cardiovascular disease, diabetic nephropathy, and diabetic retinopathy [1]. Importantly, T2DM has been reported to be involved in dementia [2]. The morbidity of cognitive impairment is particularly increased in T2DM patients older than 65 years [3]. Epidemiological study has been reported that T2DM is strongly associated with impaired cognitive function [4]. Over the past few years, the relationship between blood lipid levels and cognitive impairment has attracted more attention. It has been reported that hyperlipidemia is an independent risk factor for dementia and mild cognitive impairment in adults aged 60 years or older [5]. Lipid parameters including total cholesterol (TC), low-density lipoprotein cholesterol (LDL-C), and triglyceride (TG) have been found to associate with cognitive impairment in multiple diseases, such as patients with post-stroke and Parkinson's Disease [6, 7]. In addition, apolipoprotein B has been shown to be associated with cognitive function in patients with schizophrenia [8]. Interesting, apolipoprotein A1 has been involved in the earliest stages of Alzheimer [9]. Pillai JA found that higher plasma apolipoprotein A1 was associated with faster cognitive decline in mild cognitive impairment [10]. An association between serum apolipoprotein A1 and cognitive decline has been suggested in subjects with low brain amyloid-beta burden [11]. However, the association between blood lipid levels and cognitive impairment has not been completely studied in patients with T2DM. The present study sought to assess to clarify whether blood lipids were associated with cognitive impairment in patients with T2DM.

## Methods

### Study population

This is a cross-sectional study, a total of 336 patients with T2DM at Department of Endocrinology and Metabolism, Affiliated Zhongda Hospital of Southeast University were included  from 2020 through 2022.The diagnosis of T2DM was defined according to the American Diabetes Association criteria [12]. All patients with T2DM were classified into patients with cognitive impairment group and patients without cognitive impairment group according to the assessment of cognitive function. The diagnosis of stroke was based on magnetic resonance imaging examination in patients with T2DM. Patients with T2DM who had stroke, mental illness, systemic autoimmune disease, liver dysfunction, and cancer were excluded. In addition, we excluded T2DM patients with history of lipid-lowering drug use, and also excluded T2DM patients who were unable to complete the assessment of cognitive function. The study protocol was approved by the Ethics Committee of Zhongda Hospital, Southeast University, the processes of the study conformed to the Declaration of Helsinki, and the study obtained informed consent from all patients.

### Clinical data and cognitive function assessment

Clinical data were collected from T2DM patients. Fasting blood samples were collected for laboratory tests, including TC, high-density lipoprotein cholesterol (HDL-C), LDL-C, TG, apolipoprotein A1, apolipoprotein B, uric acid, creatinine, and glycated hemoglobin. The data in gender, age, body mass index (BMI), smoking history, drink history, disease history, education years were collected from patients with T2DM. All T2DM patients completed the Mini-Mental State Examination (MMSE) and Montreal Cognitive Assessment (MoCA) to assess the cognitive function, the MoCA assessment for cognitive impairment is more sensitive than MMSE, thus, we used MoCA to determine the cognitive impairment in patients with T2DM, the MoCA < 26 was defined as points cognitive impairment [13, 14].

### Statistical analysis

The statistical analyses were performed by the SPSS 25.0 software. Continuous variables for normally distributed variables are expressed as mean ± standard deviation, continuous variables for non-normally distributed variables are expressed as median (interquartile range), and categorical variables are expressed as percentage. Student’s *t* test or non-parametric Mann–Whitney *U* test was selected to compare the statistical differences in continuous variables as appropriate. Spearman or Pearson correlation analysis was selected to examine the correlation between the two continuous variables when  appropriate. The possible influences for independent variable were analyzed by simple logistic regression analysis, and potential confounding factors were adjusted in multivariate logistic regression analysis. A *p* value < 0.05 was regarded as significant.

## Results

The baseline characteristics of T2DM patients with and without cognitive impairment are summarized in Table 1. There were significantly differences for sex, age, education years, and glycated hemoglobin between T2DM patients with and without cognitive impairment (*p* < 0.05). Expectedly, both MMSE score and MoCA score were significantly decreased in T2DM patients with cognitive impairment compared with T2DM patients without cognitive impairment (*p* < 0.001). Serum apolipoprotein A1 levels were found to be significantly increased in T2DM patients with cognitive impairment compared with T2DM patients without cognitive impairment (*p* = 0.017).

**Table 1:** The baseline characteristics in T2DM patients with and without cognitive impairment

	Patients with cognitive impairment	Patients without cognitive impairment	*p* value
	*N* = 94	*N* = 242	
Male (*n*, %)	39 (41.5)	165 (68.2)	< 0.001
Age (years)	67 (62–73)	60 (52–66)	< 0.001
Body mass index (kg/m^2^)	24.3 (22.5–26.5)	24.9 (22.9–27.7)	0.166
Hypertension history (*n*, %)	67 (71.3)	151 (62.4)	0.126
Smoking history (*n*, %)	24 (25.5)	68 (28.1)	0.636
Drink history (*n*, %)	11 (11.7)	28 (11.6)	0.973
Education years	11 (8–14)	15 (11–15)	< 0.001
MMSE score	23 (21–24)	29 (28–30)	< 0.001
MoCA score	24 (21–25)	29 (28–30)	< 0.001
High-density lipoprotein cholesterol (mmol/L)	1.12 (1.00–1.43)	1.17 (1.01–1.36)	0.836
Low-density lipoprotein cholesterol (mmol/L)	2.32 (1.69–2.92)	2.39 (1.82–3.00)	0.275
Triglyceride (mmol/L)	1.24 (0.91–1.94)	1.41 (0.96–2.13)	0.288
Total cholesterol (mmol/L)	4.13 (3.45–4.91)	4.24 (3.33–5.07)	0.376
Apolipoprotein A1 (g/L)	1.15 ± 0.21	1.09 ± 0.20	0.017
Apolipoprotein *B* (g/L)	0.82 (0.68–0.96)	0.85 (0.69–1.02)	0.342
Apolipoprotein A1/Apolipoprotein B ratio	1.40 (1.14–1.74)	1.28 (1.04–1.65)	0.070
Uric acid (µmol/L)	283 (235–353)	300 (228–373)	0.586
Creatinine (µmol/L)	64 (54–79)	65 (55–80)	0.538
Glycated hemoglobin (%)	9.50 (8.20–10.56)	8.11 (6.80–9.90)	< 0.001

The correlations of the MoCA and MMSE score with clinical parameters are presented in Table 2. Both MoCA score and MMSE score were significantly apolipoprotein A1 (r = − 0.132, *p* = 0.016; r = − 0.143, *p* = 0.009) in patients with T2DM. Besides, the MoCA score was significantly correlated with age (*r* = − 0.511, *p* < 0.001), BMI (*r* = 0.108, *p* = 0.0.048), education years (*r* = 0.395, *p* < 0.001), apolipoprotein A1/apolipoprotein B ratio (*r* = − 0.111, *p* = 0.042), and glycated hemoglobin (*r* = − 0.248, *p* < 0.001) in patients with T2DM. The MMSE score was significantly correlated with age (*r* = − 0.442, *p* < 0.001), BMI (*r* = 0.134, *p* = 0.014), education years (*r* = 0.338 *p* < 0.001), apolipoprotein A1/apolipoprotein *B* ratio (*r* = − 0.109, *p* = 0.046), and glycated hemoglobin (*r* = − 0.235, *p* < 0.001) in patients with T2DM.

**Table 2:** The correlation between cognitive function and clinical variables in patients with T2DM

	MoCA score		MMSE score	
	*R*	*p* value	*R*	*p* value
Age	− 0.511	< 0.001	− 0.442	< 0.001
Body mass index	0.108	0.048	0.134	0.014
Education years	0.395	< 0.001	0.338	< 0.001
High-density lipoprotein cholesterol	− 0.032	0.556	− 0.008	0.888
Low-density lipoprotein cholesterol	0.044	0.426	0.042	0.448
Triglyceride	0.105	0.055	0.077	0.161
Total cholesterol	0.046	0.404	0.037	0.496
Apolipoprotein A1	− 0.132	0.016	− 0.143	0.009
Apolipoprotein *B*	0.051	0.349	0.033	0.542
Apolipoprotein A1/Apolipoprotein *B* ratio	− 0.111	0.042	− 0.109	0.046
Uric acid	0.062	0.257	0.008	0.880
Creatinine	0.020	0.717	0.003	0.962
Glycated hemoglobin	− 0.248	< 0.001	− 0.235	< 0.001

We further assessed the risk factors associated with cognitive impairment in patients with T2DM, the possible confounding factors were adjusted for sex, age, BMI, education years, smoking history, drink history, hypertension history, and glycated hemoglobin in multivariable regression model (Table 3), the results showed that serum apolipoprotein A1 was independently associated with cognitive impairment in patients with T2DM (OR = 5.201, *p* = 0.024); in addition, we also found that sex (OR = 0.370, *p* = 0.004), age (OR = 1.104, *p* < 0.001), smoking history (OR = 52.707, *p* = 50.016), education years (OR = 0.851, *p* = 0.001), and glycated hemoglobin (OR = 1.391, *p* < 0.001) independently contributed to the cognitive impairment in patients with T2DM.

**Table 3:** The assessment of logistic regression analysis for the association between blood lipid levels and cognitive impairment in patients with T2DM

	Univariable analysis		Multivariable analysis	
	OR (95%CI)	*p* value	O*R* (95%CI)	*p* value
Male	0.331 (0.202–0.541)	< 0.001	0.370 (0.186–0.734)	0.004
Age	1.099 (1.066–1.132)	< 0.001	1.104 (1.064–1.145)	< 0.001
Body mass index	0.958 (0.892–1.029)	0.237	0.977 (0.894–1.068)	0.605
Hypertension history	1.495 (0.892–2.508)	0.127	1.326 (0.689–2.550)	0.398
Smoking history	0.877 (0.510–1.508)	0.636	2.707 (1.202–6.101)	0.016
Drink history	1.013 (0.482–2.127)	0.973	2.016 (0.755–5.380)	0.162
Education years	0.816 (0.758–0.877)	< 0.001	0.851 (0.775–0.933)	0.001
Apolipoprotein A1	4.218 (1.281–13.892)	0.018	5.201 (1.240–21.822)	0.024
Glycated hemoglobin	1.247 (1.106–1.404)	< 0.001	1.391 (1.194–1.619)	< 0.001

## Discussion

Apolipoprotein A1 is a main component of high-density lipoprotein cholesterol. Increased serum apolipoprotein A1 levels have been reported in patients with Alzheimer’s disease [15]. Deng et al. [7] found that serum apolipoprotein A1 levels were significantly elevated in Parkinson’s disease patients with mild cognitive impairment, and that serum apolipoprotein A1 levels were significantly negatively associated with MoCA score in patients with Parkinson’s disease. Our study initiated to explore the relationship between blood lipids and cognitive impairment in patients with T2DM, we found that increased serum apolipoprotein A1 levels were significantly  associated with cognitive impairment in patients with T2DM.

The mechanism of this association between apolipoprotein A1 and cognitive impairment may not be completely clear in T2DM, however, some emerging evidences may contribute to explain this underlying mechanism. Demeester et al. [16] observed an increased cerebrospinal fluid apolipoprotein A1 concentrations in patients with dementia. After peripheral nerve injury, apolipoprotein A1 concentrations are significantly elevated compared with normal nerves [17]. These studies suggest that increased apolipoprotein A1 may be associated with nerve damage. Apolipoprotein A1 mimetic peptide can inhibit amyloid beta deposition and improve cognitive function by its anti-inflammatory properties in the brain [18].  The overexpression of human apolipoprotein A1 in the circulation may preserve cognitive function in patients with dementia partly by attenuating neuroinflammation and cerebral amyloid angiopathy [19]. The peripheral apolipoprotein A1 from the liver and intestine is found to enter into the central nervous system primarily by crossing the blood–cerebrospinal fluid barrier [20]. Similar reportedly, plasma apolipoprotein A1 can be transported into nerve to regenerate damaged nerves and myelin [17]. Clearly, increased lipoprotein A1 in circulation can enter the nervous system by a specific way, which may be helpful for the repairment and regeneration of damaged nerves. Interesting, the central nervous system has been reported to communicate with the liver to regulate the hepatic lipid metabolism and lipoprotein production by a bidirectional manner [21]. Thus, we deduce that the damaged central nerves may communicate with the liver to synthesize more lipoprotein A1 for its repairment and regeneration, and then abundant serum apolipoprotein A1 is transported to the central nervous system as a protective role against the central neuropathy in T2DM patients with cognitive impairment.

In the study, it was not unexpected that age and education years were significantly associated with cognitive impairment in patients with T2DM. In addition, we confirmed that gender had an association with cognitive impairment in patients with T2DM, the results imply female may be an independent risk factor for cognitive impairment in T2DM patients. Although there is inconsistency for the gender differences for the incidence rate of cognitive impairment, however, more studies have reported a higher prevalence of cognitive impairment in women [22–24]. Our study also showed that smoking and glycated hemoglobin were strongly associated with cognitive impairment in patients with T2DM, the results are consistent with previous reports [25, 26].

The limitations of the study are as follows: First, the causality between serum apolipoprotein A1 and cognitive impairment in patients with T2DM is unclear due to the cross-sectional design of this study, therefore, the interpretation of the results should be done with caution. Second, only a single levels of blood lipids were measured, so that we didn't confirm the change of apolipoprotein A1 over time in patients with T2DM. Third, the current study has no the follow-up data to determine whether baseline apolipoprotein A1 levels is a conceivable predictor for the incidence of dementia in patients with T2DM. Fourth, the current results are only limited to patients with T2DM, thus, they may be cannot be generalized to other populations.

## Conclusion

Our study assessed the association between blood lipids and cognitive function in patients with T2DM, we found that serum apolipoprotein A1 was independently associated with cognitive impairment in patients with T2DM, but not TC, TG, HDL-C, LDL-C, and apolipoprotein B. The current findings suggest increased serum apolipoprotein A1 may be considered as a risk factor of cognitive impairment in patients with T2DM.

## Data Availability

The data can be obtained from the corresponding author upon reasonable request.
